# Surveillance of Highly Pathogenic Avian Influenza Virus in Wild Canids from Pennsylvania, USA

**DOI:** 10.3390/ani14243700

**Published:** 2024-12-22

**Authors:** Kevin D. Niedringhaus, Taylor C. Chan, Ashley McDowell, Lauren Maxwell, Madison Stevens, Lane Potts, Erica Miller, Eman Anis, Kyle Van Why, Thomas Keller, David Stallknecht, Rebecca L. Poulson, Kaitlyn Bahrs, Justin D. Brown

**Affiliations:** 1 Wildlife Futures Program, School of Veterinary Medicine, University of Pennsylvania, Kennett Square, PA 19348, USA; tcychan@vet.upenn.edu (T.C.C.); ashley.mcdowell53@yahoo.com (A.M.); lmaxwel@vet.upenn.edu (L.M.); stemad@vet.upenn.edu (M.S.); lpotts@vet.upenn.edu (L.P.); millerer@upenn.edu (E.M.); eanis@vet.upenn.edu (E.A.); 2 United States Department of Agriculture, Animal and Plant Health Inspection Service, Wildlife Services, Harrisburg, PA 17106, USA; kyle.r.vanwhy@usda.gov; 3 The Pennsylvania Game Commission, Harrisburg, PA 17110, USA; thkeller@pa.gov; 4 Southeastern Cooperative Wildlife Disease Study, College of Veterinary Medicine, University of Georgia, Athens, GA 30602, USA; dstall@uga.edu (D.S.); rpoulson@uga.edu (R.L.P.); 5 Department of Veterinary and Biomedical Sciences, Pennsylvania State University, University Park, PA 16802, USA; kfb5585@psu.edu (K.B.); jdb56@psu.edu (J.D.B.)

**Keywords:** canid, coyote, encephalitis, fox, highly pathogenic avian influenza, serology, wildlife disease

## Abstract

The avian influenza virus is a global pathogen with significant health and economic implications. While primarily a pathogen of wild and domestic birds, recent outbreaks of the H5N1 highly pathogenic avian influenza virus (HPAIV) clade 2.3.4.4b have caused mortality in a wide variety of mammals, including members of the Canidae family, on multiple continents. Despite sporadic mortality events globally, the epidemiology and pathobiology of H5N1 HPAIV in wild canids remains poorly defined. During 2022–2024, 41 wild canid carcasses (diagnostic cases), including 23 red foxes and 18 gray foxes, were tested for the influenza A virus (IAV) via PCR, with five red fox kits testing positive (12%). Infected animals had variably severe encephalitis, pneumonia, and occasionally myocarditis associated with strong immunolabeling for IAV. Serum from 269 wild canids in Pennsylvania was tested for antibodies to IAV, including 133 samples collected prior to 2021 (pre-H5N1 HPAIV 2.3.4.4b introduction) and 136 collected after 2022 (post-H5N1 HPAIV 2.3.4.4b introduction). All samples collected prior to 2021 were seronegative for IAV. Two coyotes from 2024 were seropositive for IAV but were negative for antibodies to the H5 and N1 subtypes. Collectively, these data suggest that while sporadic H5N1 HPAIV infection and mortality can occur in wild canids, particularly juvenile red foxes, infection was limited in these outwardly healthy and opportunistically sampled animals. Future studies should utilize a risk-based approach to target sampling of wild canids at increased risk for H5N1 HPAIV infection, such as those around waterfowl habitats or spatially around wild bird or domestic animal outbreaks.

## Simple Summary

While wild canids are known to develop severe disease when infected with the H5N1 highly pathogenic avian influenza virus (HPAIV) clade 2.3.4.4b, infection and mortality are sporadic and typically involve individual animals. Interpretation of sporadic mortality events is challenging due to the lack of knowledge on H5N1 HPAIV ecology in wild canids and limited active surveillance data on viral infection, transmission, and/or exposure. In this study, we summarize the diagnostic data for wild canids from Pennsylvania who died and were tested for HPAIV during 2021–2024. In addition, we report serologic data from wild canids that were tested for exposure to the influenza A virus (IAV). Collectively, these data suggest that wild canids (especially young red foxes) are susceptible to H5N1 HPAIV clade 2.3.4.4b and infection can result in severe disease; however, IAV exposure in wild canids in Pennsylvania seems to be rare. None of the opportunistically collected animals tested positive for antibodies consistent with previous infection with H5N1 HPAIV. Targeting canids at increased risk of infection (e.g., those exposed to wild waterfowl or located in an area of an active outbreak in wild or domestic animals) may yield a higher seroprevalence.

## 1. Introduction

Since emerging in 1996, the goose/Guangdong (Gs/GD) lineage of the H5 highly pathogenic avian influenza virus (HPAIV) has evolved into multiple distinct groups of viruses [1,2]. The 2.3.4.4b clade has resulted in widespread mortality of domestic and wild avian species across the globe [3]. This clade was first detected in North America in late 2021 in Eastern Canada and to date (July 2024) has continued to circulate in wild and domestic birds. The only previous outbreak of the Gs/GD lineage of H5 HPAIV in North America was in 2014–2015, which was caused by H5 HPAIV clade 2.3.4.4c. The current clade 2.3.4.4b outbreak in North America differs significantly from the 2.3.4.4c outbreak, with differences including longer duration, being more geographically widespread, larger involvement of wild birds, and increased infections and mortalities in wild and domestic mammals [4,5].

Certain taxa of wild mammals appear to be particularly susceptible to disease and mortality from the H5N1 HPAI virus clade 2.3.4.4b, with population-level impacts being of significant concern [6,7,8,9]. Most of the wild terrestrial mammalian mortality events involve carnivore or omnivore species and typically result in isolated or individual mortalities. Presumably, the increased occurrence in these species partially relates to predation and/or scavenging of infected avian prey. Interpretation of the significance of these mortality events is challenging due to the lack of active surveillance data and limited knowledge of the pathobiology and epidemiology of H5N1 HPAIV in wild mammals. Specifically, it is unknown how common H5N1 HPAIV infection is in the absence of severe disease or mortality.

Sporadic and rare mortality associated with H5N1 HPAIV clade 2.3.4.4b infection has been reported in domestic dogs and some wild canid species. Domestic dog fatal H5N1 HPAIV infections were reported in Thailand [10] and Ontario, Canada [11]. Both cases involved individual dogs that had a recent history of close exposure to sick or dead waterfowl and experienced acute disease characterized by pulmonary edema and interstitial pneumonia, with necrotizing hepatitis in one dog [10] and suppurative pleuritis and fibrinonecrotizing tracheobronchial lymphadenitis in the other [11]. Dogs have also been experimentally challenged with H5N1 HPAIV clades 2.2 [12], 1.1.2, and 2.3.2.1c [13]. In these settings, the severity of clinical disease was variable, but infection consistently resulted in pneumonia. In these dogs of mixed ages, infected both naturally and experimentally, overt encephalitis or neurologic signs were not described.

While domestic dogs have been serologically surveyed for exposure to IAV, and specifically the canine influenza virus (H3N2 and H3N8), there is limited published serologic data on infection to the Gs/GD lineage of H5 HPAIV. A serosurvey of domestic dogs did not specifically detect antibodies to the H5 IAV subtype in Italy [14], Thailand [15], or South Korea [16], but a low seroprevalence (1.3%) was detected to the H5N8 subtype in dogs from the Netherlands [17]. Additional studies detected low seroprevalence for IAV in dogs from Germany (0.9%; [18]), the USA (3.5%; [19]), Poland (2.2%; [20]), and Ukraine (5.7%; [21]) via Enzyme-Linked Immunosorbent Assay (ELISA). Positive samples from these studies, when further characterized, were predominately H1 and H3 subtypes; however, the H5 subtype was not specifically investigated, so potential seropositive individuals for this subtype could not be excluded. More recently, waterfowl and upland game bird hunting dogs in Washington State were tested for antibodies to the H5 and N1 subtypes of IAV in 2023–2024, which identified a low prevalence of antibodies (2%) despite a high level of infection risk from hunting and training with birds [22]. Collectively, these studies suggest domestic dogs are susceptible to H5N1 HPAIV and severe disease is possible; however, infection is rare and typically associated with high infection risks. In wild canids, serological studies for H5 HPAIV are also rare. Seroprevalence of red fox (*Vulpes vulpes*) to 2.3.4.4 H5-HA1 in the Netherlands during the H5N1 HPAIV outbreak was 37% and showed a significantly higher number of seropositives from 2020 to 2022 compared to 2017 to 2019 [23].

Red fox kit mortalities associated with H5N1 HPAIV have been reported throughout North America, with the most common lesions being necrotizing meningoencephalitis, interstitial pneumonia, and myocardial necrosis [24,25]. These field findings align with mortality reports from Europe and experimental data indicating that the red fox is highly susceptible to Gs/GD H5 HPAIV and experiences severe disease characterized by encephalitis, interstitial pneumonia, and myocarditis [26,27]. In addition to red fox, H5N1 HPAIV has been detected by polymerase chain reaction (PCR) in a single coyote (*Canis latrans*) and gray fox (*Urocyon cinereoargenteus*), but no other wild canid mortalities have been reported in North America to date [24,28]. Currently, there is limited active surveillance data on H5 HPAIV in outwardly healthy wild canids in North America, targeting active infections or antibody presence, which significantly complicates the interpretation of these mortality events. Collectively, these results indicate that wild and domestic canids are susceptible to H5N1 HPAIV, but there is a wide range of associated disease severity within the taxa.

The increased involvement of wild mammals in the global H5N1 HPAIV outbreak has resulted in new challenges related to surveillance and management. Developing effective wildlife management programs for H5N1 HPAIV is hindered by a lack of surveillance data to provide perspective for interpreting the sporadic H5N1 HPAIV wild mammal mortality events. The highly variable, and often undefined, susceptibility and pathobiology for different wildlife species creates a challenge when using traditional surveillance methods. To address these gaps, this study utilizes a multifaceted surveillance approach by employing a combination of mortality investigations and serologic testing of opportunistically collected serum samples to survey for H5N1 HPAIV in wild canids in Pennsylvania from 2021 to 2024. Historical data were also included to compare trends before and after the introduction of H5N1 HPAIV into North America.

## 2. Materials and Methods

Blood samples were obtained opportunistically (i.e., collected without regard to HPAIV risk factors) from hunter-harvested wild canids (red fox, gray fox, and coyote) throughout Pennsylvania. Blood was collected in non-heparinized blood tubes and stored on ice prior to centrifugation and freezing before testing. None of the hunters reported that the animals were showing overt disease or abnormal behavior before harvest. The samples were centrifuged to retrieve serum and dichotomized into either pre- (2020 and older) or post- (2022–2024) H5N1 HPAIV 2.3.4.4b outbreak in North America. All work was approved by the Penn State University Biosafety Committee (BIO202300155) and the Pennsylvania Game Commission (special use permit #58351).

All serum samples were initially tested for antibodies to IAV nucleoprotein (NP) using commercial blocking Enzyme-Linked Immunosorbent Assay (bELISA; MultiS-Screen Ab Test, IDEXX, Westbrook, ME, USA), following the manufacturer’s instructions. This serologic assay identifies antibodies to IAV regardless of subtype or pathotype. Based on the manufacturer’s instructions, any sample with a serum sample to negative control absorbance ratio (S/N) ≤ 0.50 is considered positive for antibodies to the NP of IAV. Samples positive on the bELISA were subsequently tested for H5 antibodies using hemagglutination inhibition (HI) assay and virus neutralization (VN), and N1 antibodies were tested using an enzyme-linked lectin assay (ELLA), according to published protocols [29,30]. To increase the potential detection of H5 and N1 antibodies, any samples in this screening serological test with a S/N < 0.70 were further tested for H5 and N1 antibodies. The HI and VN assays were performed using two reverse genetics (RG)-derived antigens to detect antibodies to clade 2.3.4.4b H5 and North American H5 low pathogenic (LP) H5 IAV. Antigens included IDCDC-RG71A, containing both Eurasian HA and NA from A/Astrakhan/3212/2020(H5N8) on an A/Puerto Rico/8/1934(H1N1)(PR8) backbone and LP-RGBWT/TX that included the North American HA and NA from A/Blue-winged teal/AI12-4150/Texas/2012(H5N2) on a PR8 backbone. The N1 ELLA utilized the antigen A/ruddy turnstone/New Jersey/AI13-2948/2013(H10N1). Diagnostic thresholds for positive test results were based on conservative titers and included H5 HI (>1:32), H5 VN (>1:20), and N1 ELLA (>1:80).

Unless a clear cause of mortality was apparent, all wild red fox and gray fox diagnostic cases from Pennsylvania during 2022–2024 were tested for IAV (no coyotes were submitted as diagnostic cases) from an oropharyngeal swab, trachea, brain, and/or lung tissue. These diagnostic cases were submitted from throughout Pennsylvania by private citizens or wildlife rehabilitation facilities reporting sick or dead animals and were examined at the University of Pennsylvania School of Veterinary Medicine’s Wildlife Futures Program within the American Association of Veterinary Laboratory Diagnosticians (AAVLD)-accredited Pennsylvania Animal Diagnostic Laboratory System. The animals received full or partial necropsy if the entire carcass was not submitted. Age was classified as ‘juvenile/kit’ or ‘adult’ based on fur color and morphology and overall size. Other ancillary tests, including rabies virus testing by the fluorescent antibody test [31] and canine distemper virus testing by PCR [32], were also performed in a subset of cases.

Standardized protocols for the National Animal Health Laboratory Network (NAHLN) for IAV and the subtype H5 were utilized to identify positive cases. Nucleic acid was extracted using a commercial kit (MagMax-96 Viral RNA Isolation kit; Thermo Fisher) according to the manufacturer’s protocol. Initially, the samples were screened for IAV using RT-PCR, which targets the matrix gene [33,34]. All matrix-positive samples were subsequently tested for the H5 subtype according to National Veterinary Services Laboratory (NVSL; Ames, IA, USA) approved protocols [35]. Additionally, all IAV-positive samples were sent to NVSL for confirmatory testing and further characterization.

Any PCR-positive animals were further characterized by histopathology and occasionally IHC. During necropsy, representative tissues including the brain at minimum, and lung and other viscera when available, were fixed in 10% neutral buffered formalin. The tissues were trimmed down, processed routinely, cut at 4 µm thick sections, placed on glass slides, and stained with hematoxylin and eosin prior to examination via light microscopy. Immunohistochemical analysis for IAV was performed on various tissues from all positive cases, including brain, lung, and heart at minimum. The assay was performed at an AAVLD-accredited laboratory (Athens Veterinary Diagnostic Laboratory, Athens, GA, USA) by validated, established protocols using a polyclonal influenza A antiserum (ab20841, Abcam) diluted 1:600 and incubated for 60 min. Antigen retrieval was at pH 9.0 for 15 min at 110 °C, and enzyme blocking was performed by 3% H202 for 5 min. Blocking was carried out with a universal blocking reagent with a power block for five minutes; the link was GOAT-1 for 10 min, and staining was carried out with a warp red chromogen kit for 10 min. All cases were run with a duplicated but antibody-free tissue for negative control as well as a separate, known IAV-positive case as a positive control on a Biocare Medical IntelliPATH machine.

ArcGIS software was used to make two maps comparing the distribution of positive and negative PCR samples in wild canids as well as the distribution of serum samples opportunistically collected. Two additional maps were made to highlight the distribution of HPAIV detections in wild birds [36] as well as the number of domestic poultry affected by HPAIV detection [37].

## 3. Results

### 3.1. Serology

**Figure 1:**
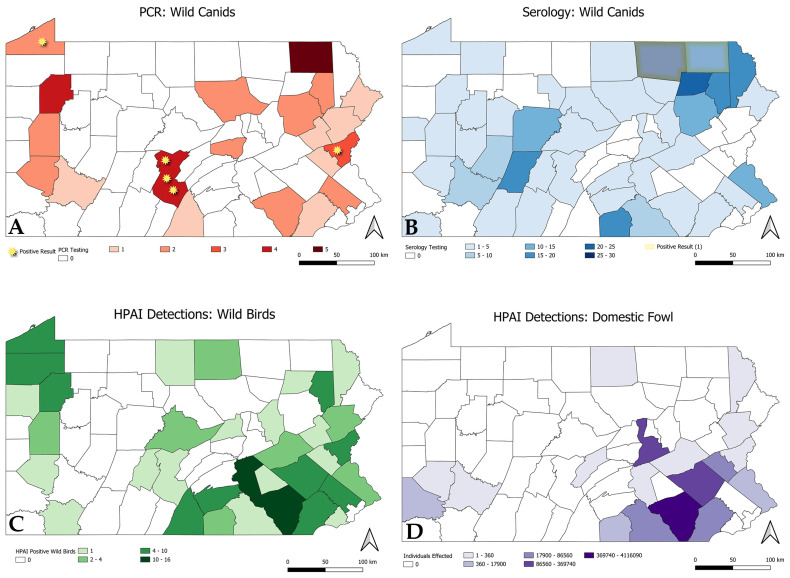
Map of Pennsylvania, USA, describing counties and quantity of serology tests in wild canids. Stars indicate positive cases by PCR. (**A**) Wild canids tested for HPAIV via PCR per county between 2022 and 2024. Positive results are indicated with a star (not to scale). There were three detections in Huntingdon County, one in Erie County, and one in Northampton County. (**B**) Wild canids tested for IAV via serology per county between 2019 and 2024. Two IAV-positive counties are highlighted. (**C**) Wild birds positive for H5N1 HPAIV by county between 2022 and 2024. (**D**) Domestic fowl with an HPAIV detection.

A total of 268 wild canid serum samples were tested for antibodies to IAV NP, including 133 collected prior to H5N1 HPAIV introduction into North America in 2021, and 135 after. Pre-outbreak samples were collected during 2019–2020 from multiple Pennsylvania counties and consisted of 82 coyotes, 10 gray foxes, and 41 red foxes. Post-outbreak samples were collected in 2024 from multiple Pennsylvania counties and consisted of 95 coyotes, 21 gray foxes, and 19 red foxes (Figure 1 and Table 1). All pre-outbreak serum samples tested negative for antibodies to IAV NP based on a diagnostic cutoff of SN value ≤ 0.5. The mean S/N value was 0.91 (range: 0.62–1.82). Six (4.5%; 6/133) serum seronegative samples had S/N between 0.5 and 0.7 and were tested for antibodies to H5 and N1, and all were negative. Two (1.4%; 2/135) post-outbreak wild canid samples (both coyotes) tested positive for antibodies to IAV NP based on a S/N value of ≤0.5, and the remaining 133 samples were seronegative (SN ≥ 0.7). The mean S/N for all post-outbreak samples was 0.82 (range: 0.49–2.00). The two samples that were positive for IAV NP antibodies both had an S/N value of 0.49. Twenty-eight seronegative samples had S/N values between 0.5 and 0.7 and were also tested for antibodies to H5 and N1. All post-outbreak serum samples tested for subtype-specific antibodies, including the two seropositive for IAV NP and the 28 with S/N values between 0.50 and 0.70, were negative for antibodies to H5 and N1.

### 3.2. Diagnostic Cases

Forty-one wild canids were tested for IAV in Pennsylvania between 2022 and 2024 (no submissions or tests recorded prior to 2022) by RT-PCR targeting the M gene [33,34]. Sample types included tracheal swabs (3), brain (17), lung (4), and pooled brain and lung (11) from 23 red foxes and 18 gray foxes. No coyote samples were received or tested during this timeframe. Between April 10 and July 2024, HPAIV was detected in five red foxes (12.2% of all canids; 21.7% of all red foxes tested). Positive samples were brain (4/5) and lung (1/5) and were confirmed by RT-PCR for H5 and N1 [35]. IAV was not detected in any gray foxes. Ct values from all tests performed at NVSL are provided in Table S2.

The positive cases originated from three counties in Pennsylvania: Huntingdon, Northampton, and Erie between April and June of 2023 (Figure 1). All positive red foxes were juveniles found alive with multiple littermates and/or dam found dead at the site of their retrieval. Live fox kits were euthanized soon after admission to various wildlife clinics or at regional government offices due to moribund conditions. All five euthanized animals exhibited neurological abnormalities comprising seizures (n = 4), blindness (n = 1), disorientation (n = 1), and recumbency (n = 1). Other clinical signs included lethargy (n = 1), dehydration (n = 1), and inappetence (n = 1). Three animals, all from the same litter, were tested for IAV via a commercial rapid FLU-detect kit at the clinic and were positive. The other two animals were not tested with this kit. See histories and clinical summaries in Table S1.

**Figure 2:**
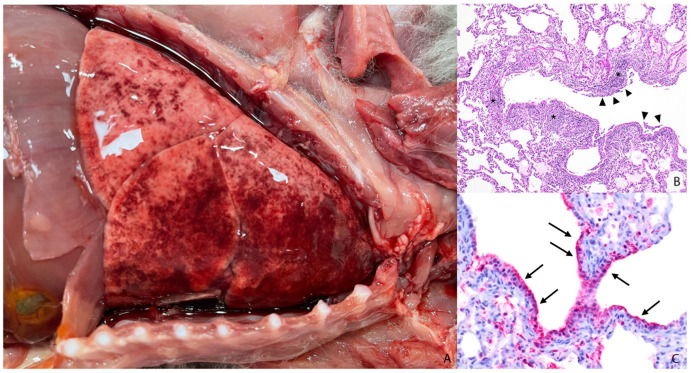
Gross and microscopic findings of pulmonary lesions in HPAIV-positive red foxes (*Vulpes vulpes*). (**A**) The lungs are non-collapsing, mottled dark red and pink with mild rib impressions. (**B**) The pulmonary interstitium surrounding the terminal bronchioles is mildly expanded by lymphocytes and macrophages (asterisk) and the epithelium is replaced by necrotic debris and fibrin (arrowheads) (determined via H&E). (**C**) The apical surface and cytoplasm of bronchiolar epithelial cells of multiple bronchioles and alveolar macrophage cytoplasm exhibit strong immunoreactivity for the influenza A virus antibody (arrows) (determined via IHC).

At postmortem evaluation, gross observations of the body were recorded in two animals, and only the brain was sampled in the remaining three animals. The animals ranged from thin to fair nutritional condition. Gross findings included bilateral ocular discharge (n = 1) and mottled red–pink lungs (n = 2; Figure 2A) with mild rib impressions (n = 1) and loose feces (n = 1). Brain tissue from all five animals was confirmed to be negative for antigens of the rabies virus by a fluorescent antibody test.

**Figure 3:**
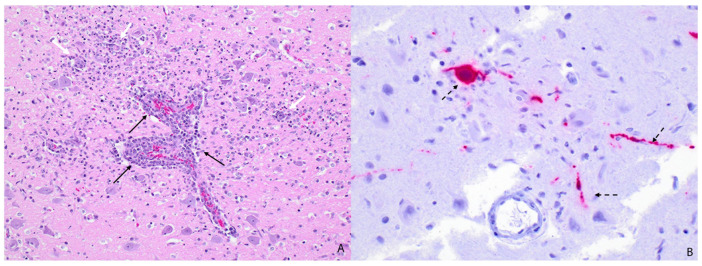
Microscopic findings of encephalitic lesions in HPAIV-positive red foxes (*Vulpes vulpes*). (**A**) Cerebrum with necrotizing encephalitis. The Virchow–Robin spaces are expanded by lymphocytes, plasma cells, neutrophils, and histiocytes (black arrows). Karyorrhectic debris and inflammatory cells surround neuronal cell bodies and infiltrate the adjacent neuropil (white arrows) (determined via H&E). (**B**) The neuronal cell bodies and processes exhibit strong intracytoplasmic immunoreactivity (dotted arrows) (determined via IHC).

Histopathologic evaluation was performed on the brain (n = 4), heart (n = 2), and lung (n = 2) among other viscera. Severe necrotizing meningoencephalitis was present in all animals with the brain available for histopathologic evaluation. Encephalitic lesions were characterized by perivascular cuffing and infiltration of lymphocytes, plasma cells, neutrophils, and histiocytes into the Virchow–Robin space and expanding the leptomeninges (Figure 3A). Fragmented inflammatory cells, karyorrhectic debris, vacuolation to malacia, individual neuronal necrosis, and glial nodules were present throughout the neuropil and affected both the grey and white matter. Neutrophilic bronchopneumonia was found in both animals and concurrent lymphohistiocytic interstitial pneumonia was found in one of the animals. Lung lesions were characterized by large numbers of neutrophils filling the large airways and extending into terminal bronchioles and the alveolar lumina. The pulmonary interstitium was mildly expanded by lymphocytes and histiocytes. The alveolar lumina frequently contained foamy alveolar macrophages, fibrin, and edema, and the alveolar walls were lined with prominent type II pneumocytes (Figure 2B). In one of the animals, scattered nematode larvae were present within the bronchi. Other less common histopathologic findings included myocardial necrosis (n = 1) and mineralization (n = 1), lymphohistiocytic myositis (n = 1), lymphocytic conjunctivitis (n = 1), and small intestinal ascariasis (n = 1).

All brain tissue exhibited moderate cytoplasmic and strong nuclear immunoreactivity in neuronal cell bodies, which extended into the neuronal processes (Figure 3B). In multiple sections of lung tissue, there is strong immunoreactivity in the apical surface and cytoplasm of bronchiolar epithelial cells of multiple bronchioles and within the cytoplasm of macrophages within the surrounding interstitium and alveoli (Figure 2C). Mild nonspecific immunoreactivity was present in the regions of myocardial necrosis and mineralization.

Examining the number of wild bird (Figure 1C) and domestic poultry (Figure 1D) detections and depopulations, respectively, most avian cases originated in the southeastern part of Pennsylvania. Specifically, Lancaster County had the highest detections in both wild and domestic birds. Dauphin and Berks Counties also reported high numbers of detections in wild birds and affected domestic birds, respectively. Canids tested by PCR were more widespread throughout the state (Figure 1A) with most samples from Susquehanna County in the northeast, Venango County in the northwest, and Huntingdon County in the southcentral regions. Most serology samples originated from the northeastern part of the state (Figure 1B) including Wyoming, Lackawanna, and Wayne Counties.

## 4. Discussion

Increased detections of H5N1 HPAIV in wild mammals in the current North American outbreak pose unique challenges for the surveillance and management of wildlife. Passive surveillance efforts have identified H5N1 HPAIV in a diversity of moribund or dead wildlife in North America since 2022. However, the lack of experimental data on H5N1 HPAIV in wild mammals and limited surveillance data on outwardly healthy animals make it difficult to interpret sporadic, individual mortality events. For example, it is unclear if increased fatal H5N1 HPAIV infections in wild carnivores and omnivores reflect increased adaptation and transmission in wildlife populations or increased infection from predation or scavenging wild or domestic birds that died of H5N1 HPAIV on the landscape. In this study, we utilized serology as a complement to the diagnostic examination of mortality events to examine H5N1 HPAIV infection in outwardly healthy wild canids.

Influenza serology surveys are comparatively less common in free-ranging canids and thus our overall understanding of this virus is limited compared to domestic dogs. In addition, relatively little is known about the risks of H5N1 HPAIV infection in wild canids. Understanding influenza infection dynamics in this group of animals is important to predict the effects on individual wildlife and population-level health, the potential for transmission between individuals and across species, and ensuring diagnostic and surveillance efforts can adequately identify and monitor infections. By utilizing both serology and postmortem diagnostics, our data show that wild canids in Pennsylvania can be infected with H5N1 HPAIV clade 2.3.4.4b, and infection can be fatal, particularly in juvenile red foxes; however, serologic data provide no evidence of widespread infection. The two seropositive coyotes had S/N values very close to the diagnostic threshold for positivity, and both were negative for H5 and N1 antibodies. These findings suggest possible exposure to IAV of other subtypes. The notable lack of seropositive canids may also reflect acute disease and mortality prior to antibody development and detection. The findings from our study conflict with a study from the Netherlands that described high (30%) seroprevalence and high (29%) antigen detection compared to 0% seroprevalence and 27% antigen detection in red foxes from our study [23]. The reason for this discrepancy is unknown but could be due to differences in risk related to opportunistic sampling of animals without regard to their proximity to waterfowl habitats or known H5N1 HPAIV outbreak areas.

Postmortem lesions in this small case series are similar to those reported in red foxes in other parts of the USA and Europe [24,27,38]. Like those studies, red fox kits appear to be more likely to develop disease due to HPAIV infection, although two scenarios in our study described an adult fox being dead when kits were collected (those animals were not available for postmortem evaluation). While gray foxes and coyotes are reported to develop morbidity and experience mortality from HPAIV infection in other states [24,28], these species were not diagnosed with HPAIV-associated mortality in Pennsylvania utilizing a passive surveillance approach. Free-ranging red foxes appear to be particularly susceptible to H5N1 HPAIV, which has shown adaptive mutations and an affinity for the nervous system [38]. The lack of mortality diagnosed in gray foxes and coyotes in Pennsylvania is unknown but both species are also comparatively less commonly diagnosed in other states [28]. The gray fox specifically showed an overall paucity of lesions in one study describing HPAI detection, suggesting that infection could have been subclinical [24].

It is important to consider HPAIV in neurologic red foxes and that detecting the virus in brain tissue may be more suitable than typical oropharyngeal or rectal swabs taken in birds [26]. Based on suspected tissue tropism and molecular epidemiology/sequencing of viruses in red foxes, virus contraction is most likely and most commonly due to the consumption of infected birds [38]. However, there is some evidence of virus shedding in red foxes surviving the infection, suggesting horizontal transmission is possible [26,38]. Two of the three mortality events in this study involved multiple animals, emphasizing the commonality of multi-animal mortality events. Similar to the rabies and canine distemper viruses, HPAIV should be considered among the diseases to test for when neurologic canids are evaluated, particularly in fox kits, as the clinical signs, as well as gross and microscopic lesions, can be very similar. Considering the similarities in the clinical presentation between rabies and HPAIV infection in red foxes, most neurologic canids are submitted to public health laboratories for rabies testing and may not undergo further testing. Coordination between diagnostic laboratories should be considered to ensure these suspect cases are not missed. Both PCR and IHC on tissue proved to be useful diagnostic tests to confirm infection with HPAIV. The study from the Netherlands also emphasizes the benefit of testing for H5 HPAIV in red foxes even in the absence of overt encephalitis, particularly if known risk factors are identified [23].

While biases in species submitted to wildlife clinics or to laboratories for postmortem examination invariably occur, our data and others suggest there is extensive variability in susceptibility to H5N1 HPAIV among canids, but red foxes may be particularly susceptible compared to gray foxes, coyotes, and wolves [24,25,39]. The reason for this is unknown and may reflect more common viral infection than other canid species, particularly related to backyard chicken flocks, their increased peri-urban lifestyle increasing the likelihood of the public recognizing sick animals, habitat use associated with more open agriculture with potential higher exposure to poultry farms or large flocking waterfowl, or may be due to particularly high virulence in this species. Kits may also be particularly predisposed to severe disease; however, the paucity of serum samples from fox kits to determine potential infection and survival in this age group hindered the ability to make more formal conclusions.

Based on serologic testing of historic and contemporary samples in this study, IAV infection with survival in wild canids in Pennsylvania was rare. None of the serum samples collected prior to H5N1 HPAIV introduction to North America were positive for IAV antibodies. Serum samples collected from two coyotes after H5N1 HPAIV introduction were positive for IAV antibodies, but both samples had S/N values close to the diagnostic threshold for positivity. Neither of these samples was positive for H5 or N1 antibodies and may reflect previous infection with LP IAV of another subtype. These data suggest that H5N1 HPAIV infection in outwardly healthy wild canids in Pennsylvania is uncommon. However, it is important to note that serum samples in this study were opportunistically collected from hunter-harvested wild canids. Wildlife surveys in game species frequently rely heavily on passive surveillance approaches, in which samples are collected from animals that are readily available, such as those harvested by hunters and delivered to check stations, dispatched for wildlife control purposes, or submitted for diagnostic investigations. Utilizing these sources allows for larger sample sizes with reduced added effort and resources beyond what action is already being conducted. However, in most instances, these sampling approaches have limited ability to target specific locations, time periods, or host demographics. This presents a significant limitation for HPAIV surveillance of wildlife, particularly in species that are poorly defined IAV hosts. Figure 1 emphasizes in our study that opportunistic sampling of canids did not directly correlate to locations of outbreaks in either poultry or wild birds, which may be a reason for low molecular and antibody detection. The spatial scales used in wildlife studies (wildlife management unit, township, or county, which was used in this study) also inhibit the ability to confidently correlate related transmission events without more rigorous and fine-scale spatial data.

Future HPAIV surveys should consider utilizing a risk-based approach and actively target sampling animals at increased likelihood for H5N1 HPAIV infection based on species, clinical signs, or temporospatial associations with outbreaks in domestic or wild birds. As Gs/GD H5 HPAIV continues to circulate in North America, the risk for the emergence of genetic and biologically novel viruses increases. Consequently, the epidemiology and pathobiology of Gs/GD H5 HPAIV in wild mammals are subject to change [5], and continued surveillance is necessary to detect future cases.

## 5. Conclusions

By utilizing opportunistic samples from hunters and diagnostic submission data, there was little evidence of widespread avian influenza virus infection in free-ranging canids in Pennsylvania. Serologic testing revealed no positive animals. Future surveillance utilizing serology for evidence of avian influenza detection in canids should be targeted to animals with a higher likelihood of positive detection including those with suspected increased contact with waterfowl (wetland areas, lowlands, floodplains, etc.) or backyard poultry. When infection occurs, clinical disease is severe, particularly in red fox kits. The effect of infection in coyotes or gray foxes is unknown in the absence of positive detections in this study.

## Figures and Tables

**Table 1:** Influenza A bELISA serology results from wild canids in Pennsylvania pre- and post-H5N1 HPAI outbreak.

	Coyote			Red Fox			Gray Fox		
Sampling Period (Year)	Juvenile	Adult	Unknown	Juvenile	Adult	Unknown	Juvenile	Adult	Unknown
Pre-outbreak (2019–2020)	0/51	0/28	0/4	0/21	0/20	-	-	0/10	-
Post-outbreak (2024)	1/47 (2.1%)	1/48 (2.0%)	-	0/8	0/11	-	0/3	0/17	-

## Data Availability

Data were obtained from a government-sanctioned diagnostic database; all relevant information was presented in this study. Data on serology were not kept due to negative results.

## Supplementary Materials

The following supporting information can be downloaded at https://www.mdpi.com/article/10.3390/ani14243700/s1: Table S1. Details of history and antemortem clinical signs and testing of young red foxes that tested positive for H5N1 HPAI. Table S2. A summary of the Ct values of various tissues from HPAIV RT-rtPCR-positive red foxes in Pennsylvania, USA.
