# Supplementary material for: Surveillance of Highly Pathogenic Avian Influenza Virus in Wild Canids from Pennsylvania, USA
Source: Animals (Basel). 2024 Dec 22;14(24):3700. doi: 10.3390/ani14243700 (PMC11672457; doi:10.3390/ani14243700)
Supplement: Supplementary file 1 [file animals-14-03700-s001.zip › animals-3340137-supplementary.pdf]

Table S1. A summary of the history and antemortem clinical signs and testing of young red foxes that tested positive for H5N1 HPAI in Pennsylvania, USA.

	Field History				Clinic History and antemortem testing		
**Case**	**County**	**Date**	**Number of animals reported dead**	**Number of animals alive and taken to wildlife clinic**	**Clinical signs**	**Mortality**	**Tested by rapid Fludetect kit**
1	Huntingdon	April 2023	1 adult	3 kits	Seizures in 2 out of 3 kits one day after admission to clinic	All 3 euthanized	All 3 kits positive
2	Northampton	April 2023	Multiple (unknown number) kits	1 kit	Emaciated, dehydrated, lethargy, blind in one eye on admittance to clinic	Euthanized	Not tested
3	Erie	May 2023	1 adult, 1 kit	1 kit	Seizures, unconscious, peddling observed by PGC staff; not taken to clinic	Euthanized	Not tested

Table S2. A summary of the Ct values of various tissues from HPAIV RT-rtPCR positive red foxes in Pennsylvania, USA.

	IAV M		IAV H5		IAV N1		H5 2.3.4.4	
**Case**	**Brain**	**Lung**	**Brain**	**Lung**	**Brain**	**Lung**	**Brain**	**Lung**
1	13.6	N/A	8.8	N/A	15.0	N/A	8.2	N/A
2	14.0	N/A	11.7	N/A	18.0	N/A	11.6	N/A
3	13.7	N/A	10.1	N/A	15.7	N/A	9.5	N/A
4	21.61	N/A	23.74	N/A	19.0	N/A	19.8	N/A
5	N/A	27.99	N/A	30.95	N/A	27.0	N/A	24.4

N/A: Not available; the tissue was not available for testing, or the National Veterinary Services Laboratory did not provide Ct values for the test.
